# The Prevalence of Porcine Circovirus-like Viruses in China Presents New Challenges to the Diagnosis of Diarrhea-Associated Viruses. Comment on Yang et al. Epidemiology and Evolution of Emerging Porcine Circovirus-like Viruses in Pigs with Hemorrhagic Dysentery and Diarrhea Symptoms in Central China from 2018 to 2021. *Viruses* 2021, *13*, 2282

**DOI:** 10.3390/v14050962

**Published:** 2022-05-05

**Authors:** Meng Zeng, Chihai Ji, Yuan Sun, Jingyun Ma

**Affiliations:** 1 College of Animal Science, South China Agricultural University, Guangzhou 510642, China; zmeng0909@163.com (M.Z.); jichihai@163.com (C.J.); 2 Guangdong Laboratory for Lingnan Modern Agriculture, Guangzhou 510642, China; 3 Key Laboratory of Animal Health Aquaculture and Environmental Control, Guangzhou 510642, China

Recently, a report in *Viruses* has highlighted the problem of porcine circovirus-like (PCL) virus [1]. PCLV is a novel circular replication-associated protein (Rep)-encoding single stranded (CRESS) DNA virus, which was first identified in swine fecal samples in the United States in 2011 [2]. PCLV is reported to be associated with porcine diarrheal disease, and there is currently a lack of effective detection methods to monitor and understand the development of this virus. 

Pigs are almost ubiquitous as farm animals and are a great source of protein. Neonatal piglets are highly susceptible to certain enterovirus infections, leading to diarrhea, which has caused significantly increased mortality and morbidity in piglets [3]. Current research has shown that PCLV mostly exists in piglets. This virus can cause diarrhea in piglets and seriously endanger the health of piglets, and it is rarely reported in mature pigs [4,5]. At present, although there is no serious outbreak of PCLV in China, it has appeared in many provinces, and its potential threat cannot be ignored. Since 2014, this virus has been reported in Sichuan province, Anhui, Guangxi, and Guangdong, China (Figure 1) [4,5]. In February 2022, we also identified PCLV from fecal swabs of 5-day-old piglets in Hunan Province (data not shown). It is worth noting that PCLV was identified for the first time in dead sows suffering from porcine epidemic diarrhea virus (PEDV) in Anhui province in 2021, providing new epidemiological information for adult pigs infected with PCLV (Figure 1) [1].

Viral diarrheal diseases caused by various swine diarrhea etiologies and co-infections with multiple viruses are very common in diarrheal pigs [2,6]. Current research has shown that PCLV virus is co-infected with other viruses, such as porcine circovirus 2 (PCV-2), porcine parvovirus (PPV) and PEDV [1,4,5]. Some co-infection cases caused the death of piglets [1]. Whether PCLV is a decisive factor in the fatal case or its presence could promote the infection and replication of other viruses and result in the death of pigs requires further verification.

Through complete genomic sequence alignment analysis, it was found that there were multiple amino acid mutations at the same site of PCLV genomes of different branches, and it was noted that a Chinese strain of PCLV showed recombination at the Rep gene [1]. Given recombination is a driving force in the evolution of viruses, it is speculated that PCLV may be in a state of continuous evolution, and the impact of this evolution is unpredictable. Future research needs to clarify the impact of mutations on virus replication and transmission mechanisms. There are also studies showing that reported PCLV genomes have high similarities (>80%) with the sequence of Bo-Circo-like virus CH, a calf diarrhea-related calf diarrhea discovered in China in 2018, and Po-Circo-like virus-21 shares the highest similarities of 93.9% with Bo-Circo-like virus CH [7]. However, the genome of the virus had only one larger ORF. This finding indicated that the virus is different from PCV and is similar to human fecal viruses [8]. These reports remind us that the prevention and control of PCLV infection will be extremely challenging if PCLV can be transmitted across species to non-porcine hosts and adapted to new species.

At present, PCLV has not been successfully isolated from cells, and it is temporarily impossible to conduct animal model tests on the pathogenicity of PCLV. Therefore, we cannot determine the factors that have influenced the evolution of PCLV viruses. Both natural selection and mutational pressures appear to influence PCLV evolution, and these factors warrant further attention. At the same time, we should speed up the research on its pathogenic mechanism and epidemic potential, and be alert to the harm caused by PCLV to the pig industry to avoid a large-scale PCL epidemic.

## Figures and Tables

**Figure 1:**
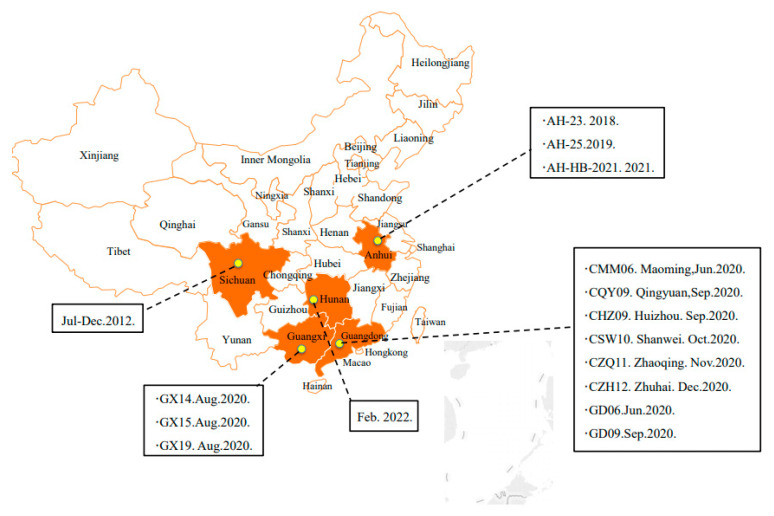
Geographic distribution of pigs with porcine circovirus-like virus in China. Provinces where the porcine circovirus-like virus has occurred are shown in orange, with detailed information, such as time, address, and virus name.

## References

[1] Yang K., Zhang M., Liu Q., Cao Y., Zhang W., Liang Y., Song X., Ji K., Shao Y., Qi K. (2021). Epidemiology and Evolution of Emerging Porcine Circovirus-like Viruses in Pigs with Hemorrhagic Dysentery and Diarrhea Symptoms in Central China from 2018 to 2021. Viruses.

[2] Shan T., Li L., Simmonds P., Wang C., Moeser A., Delwart E. (2011). The Fecal Virome of Pigs on a High-Density Farm. J. Virol..

[3] Zhang B., Tang C., Yue H., Ren Y., Song Z. (2014). Viral metagenomics analysis demonstrates the diversity of viral flora in piglet diarrhoeic faeces in China. J. Gen. Virol..

[4] Sun W., Wang W., Cao L., Zheng M., Zhuang X., Zhang H., Yu N., Tian M., Lu H., Jin N. (2020). Genetic characterization of three porcine circovirus-like viruses in pigs with diarrhoea in China. Transbound. Emerg. Dis..

[5] Liu X., Zhang X., Xu G., Wang Z., Shen H., Lian K., Lin Y., Zheng J., Liang P., Zhang L. (2021). Emergence of porcine circovirus-like viruses associated with porcine diarrheal disease in China. Transbound. Emerg. Dis..

[6] Sachsenröder J., Twardziok S., Hammerl J.A., Janczyk P., Wrede P., Hertwig S., Johne R. (2012). Simultaneous Identification of DNA and RNA Viruses Present in Pig Faeces Using Process-Controlled Deep Sequencing. PLoS ONE.

[7] Guo Z., He Q., Tang C., Zhang B., Yue H. (2018). Identification and genomic characterization of a novel CRESS DNA virus from a calf with severe hemorrhagic enteritis in China. Virus Res..

[8] Castrignano S.B., Nagasse-Sugahara T.K., Kisielius J.J., Ueda-Ito M., Brandão P.E., Curti S.P. (2013). Two novel circo-like viruses detected in human feces: Complete genome sequencing and electron microscopy analysis. Virus Res..

